# Recent Developments in Microbe–Plant-Based Bioremediation for Tackling Heavy Metal-Polluted Soils

**DOI:** 10.3389/fmicb.2021.731723

**Published:** 2021-12-23

**Authors:** Lala Saha, Jaya Tiwari, Kuldeep Bauddh, Ying Ma

**Affiliations:** ^1^Department of Environmental Sciences, Central University of Jharkhand, Ranchi, India; ^2^Department of Community Medicine and School of Public Health, PGIMER, Chandigarh, India; ^3^College of Resources and Environment, Southwest University, Chongqing, China

**Keywords:** bioremediation, beneficial microorganisms, heavy metals, phytoremediation, soil management

## Abstract

Soil contamination with heavy metals (HMs) is a serious concern for the developing world due to its non-biodegradability and significant potential to damage the ecosystem and associated services. Rapid industrialization and activities such as mining, manufacturing, and construction are generating a huge quantity of toxic waste which causes environmental hazards. There are various traditional physicochemical techniques such as electro-remediation, immobilization, stabilization, and chemical reduction to clean the contaminants from the soil. However, these methods require high energy, trained manpower, and hazardous chemicals make these techniques costly and non-environment friendly. Bioremediation, which includes microorganism-based, plant-based, microorganism-plant associated, and other innovative methods, is employed to restore the contaminated soils. This review covers some new aspects and dimensions of bioremediation of heavy metal-polluted soils. The bioremediation potential of bacteria and fungi individually and in association with plants has been reviewed and critically examined. It is reported that microbes such as *Pseudomona*s spp., *Bacillus* spp., and *Aspergillus* spp., have high metal tolerance, and bioremediation potential up to 98% both individually and when associated with plants such as *Trifolium repens, Helianthus annuus*, and *Vallisneria denseserrulata*. The mechanism of microbe’s detoxification of metals depends upon various aspects which include the internal structure, cell surface properties of microorganisms, and the surrounding environmental conditions have been covered. Further, factors affecting the bioremediation efficiency and their possible solution, along with challenges and future prospects, are also discussed.

## Introduction

With the onset of the twentieth century, human beings have witnessed advancement in technologies related to food production, health, infrastructure, transport, and communications. Such activities require a vast quantity of new materials and energies destroying natural environmental components and the production of huge quantities of wastes resulting in environmental degradation (Mani and Kumar, 2014). The presence of toxic metals and metalloids in the waste generated from the industrial, domestic, and agricultural sectors causes significant damages to the ecosystem and associated lives (Pourret et al., 2016; Goyal et al., 2020; Leong and Chang, 2020). The contaminants are highly mobile and soluble, thus possessing the capability to be bioaccumulated in the food chain and causing serious damage with increasing tropic levels (Petavratzi et al., 2005; Zerizghi et al., 2020). When these contaminants enter the human body, they can cause various life-threatening diseases such as cancer, kidney and bone diseases, cardiovascular diseases, hypertension, low birth weight, Alzheimer diseases, and atherosclerosis (Nawrot et al., 2006; Ahern et al., 2011; Bernhoft, 2012; Flora et al., 2012; Muszynska and Hanus-Fajerska, 2015; Lee et al., 2017). Metal accumulated in biological tissues is hard to remove due to its non-biodegradability, and it becomes a major concern to global health (Ayangbenro and Babalola, 2017). Metal contamination leads to the alteration in soil physicochemical and biological properties such as an increase in bulk density and soil pH, as well as a decrease in soil fertility and water holding capacity, microbial diversity and soil enzyme activity (Wuana and Okieimen, 2011; Jin et al., 2019; Saha and Bauddh, 2020). They are also responsible for the alteration in microbial communities, leading to disturbing the proper function of the biogeochemical cycle and imbalance in the ecosystem (De Quadros et al., 2016; Feng et al., 2019). Heavy metals like As, Hg, Ni, Cr, Pb, and Cu can cause multiple indirect and direct effects on plant growth, such as chlorosis, necrosis, root injury, reduced carotenoid concentration, oxidative stress, inhibition of enzyme activities, osmotic imbalance, decreased photosynthetic activities, and imbalance of the nutrients (Lewis et al., 2001; Mascher et al., 2006; Shaibur et al., 2009; Yadav, 2010; Hasan et al., 2017; Sachan and Lal, 2017). Further, due to these environmental effects of metals, there are incessant efforts made to sustainably eliminate this toxic and excess amount of metals for stabilizing the ecosystem.

Various physicochemical techniques (such as extraction, immobilization, stabilization, coagulation, electrodialysis, vitrification, reverse osmosis, ion exchange, chemical reduction, evapotranspiration, and precipitation) have already been practicing to reduce metal contamination (Ali et al., 2013; Gupta and Kumar, 2017). However, these techniques are costly, require high energy, harsh chemicals with low removal efficiency, and can generate secondary environmental pollution (Tang et al., 2007; Acheampong et al., 2010; Ali et al., 2013; Gupta and Diwan, 2016; Suman et al., 2018). Therefore, there is a continuous demand for environmental friendly remediation methods that can be helpful to reduce its harmful effects on the environment.

Bioremediation is an ecologically sound technique that requires the use of green plants, microorganisms including fungi, bacteria, yeast, and algae or their enzymes to help the polluted sites return to their original states (Chakraborty et al., 2012; Mani and Kumar, 2014). The late 19^th^ century ascertained to be the golden period for bioremediation. With further improvement, the 20^th^ century marked the beginning of research in the field of microbial ecology, involving the identification and isolation of microbes that have the potential to degrade pollutants, e.g., *Candidatus accumulibacter* that is capable of accumulating excess amount of phosphorus as polyphosphates in their cells from the sewage treatment plants (Seviour et al., 2003). Later, the delineation of catabolic pathways to break pollutants, the genomic construction of recombinant microbes tailored to eliminate metals, and the application of molecular techniques to understand microbial activities have been explored (Siezen and Galardini, 2008; Ramos et al., 2011).

Soil microorganisms play an essential role in stabilizing soil macroaggregates by producing polysaccharides to maintain soil architectural patterns for plant productivity (Ghose, 2005). Such microorganisms including numerous species of bacteria, fungi, yeast, and algae contribute significantly to the decomposition and stabilization of inorganic and organic pollutants (Fulekar et al., 2012; Rahman et al., 2015; Leong and Chang, 2020). A number of studies have highlighted that various natural and genetically engineered microorganisms (GEM) such as *Bacillus cereus*, *Chlorella pyrendoidosa, B. cereus* XMCr-6, *Pseudomonas veronii* 2E*, P. aeruginosa, Serratia marcescens, Sacharomyces cerevisiae, Penicillium canescens Spirogyra* sp., *Spirullina* sp., and *Cladophora* sp. are responsible to remediate HMs such as Cd, Pb, As, Cr, Mn, Cu, U, Se, and Zn from contaminated land and water (Lee and Chang, 2011; Kumar et al., 2011b; Hrynkiewicz et al., 2012; Kanmani et al., 2012; Mane and Bhosle, 2012; Mani and Kumar, 2014; Farhan and Khadom, 2015; Lívia et al., 2015; Ojuederie and Babalola, 2017; Verma and Kuila, 2019).

There is a need for characterization and regular assessment of various contaminated sites such as mining dumpsites, nuclear waste, surface wastewater, sewage sludge pump sites, agricultural soils, and various industrial and commercial dumping zones. Recently a number of research studies and literature reviews have been focused on the phytoremediation potential of particular plant species and selected metals with different microorganisms or particular microorganism-based remediation strategies (Raza et al., 2020; Yan et al., 2020; Wang et al., 2021; Hao et al., 2021; Sharma et al., 2021).

In this review, we have covered some new aspects and dimensions of bioremediation of heavy metal-polluted soils. Here, we have reviewed the recent literature published mainly between the year 2019–2021. There is a critical examination of the bioremediation potential of different microorganisms, especially bacteria and fungi individually and in association with plants. Further, the different mechanisms adopted by the microorganisms to detoxify HMs have also been discussed. Moreover, the study attempts to explore the knowledge about field applications with several case studies, factors affecting bioremediation, challenges, as well as future prospects have been covered.

## Methodology

The relevant literature was searched and collected from the online database using Scopus, Web of Science, Google, Google Scholar, Springer Nature, Frontiers, Taylor and Francis, Science Direct, etc. The keywords used for the literature search include bioremediation, phytoremediation, phytoextraction, phytomanagement, remediation using living organisms, remediation through plant/microorganism, plant–microbe association for heavy metal removal, etc. In addition, particular focus journals such as International Journal of Phytoremediation, Bioremediation Journal, Frontiers in Microbiology, Journal of Environmental Management, Frontiers in Plant Science, Science of the Total Environment, Chemosphere, Water, Air, & Soil Pollution, Environmental Science and Pollution Research, Microbial Research, etc. were browsed volume-wise for track the relevant papers until July 2021. The literature includes journal articles, books, book chapters, conference papers, proceedings, and technical reports were referred in this review paper from which 92.91% were published between the years 2010 to 2021. In total, more than 400 documents were examined individually and eliminated the quotative and duplicate papers (Qi et al., 2018). Out of which 254 documents were selected for reference in this work.

## Bioremediation

Bioremediation is an emerging and highly acceptable practice for restoring heavy metal contaminated soils, because of its environment friendly and low cost as compared to other conventional methods such as dredging, capping, and incineration that are often very costly and ineffective when metal concentration level is low and often generates a significant amount of toxic byproducts (Ekperusi and Aigbodion, 2015; Ayangbenro and Babalola, 2017). A study has been shown that it costs about 100–500 USD/ton for cleaning metal-polluted sediments and soils through landfilling and chemical treatment, and 90–870 USD/ton for vitrification, whereas about 15–200 USD/ton for bioremediation and 5–40 USD/ton for phytoremediation (Meier et al., 2012). It estimates that bioremediation can save 50–65% for cleaning one acre of Pb-contaminated soil compared to traditional excavation and landfill (Blaylock et al., 1997; Chibuike and Obiora, 2014). In addition, bioremediation is a non-invasive method that can remove contaminants permanently, leave the environment intact, and can be hybridized with chemical and physical treatments (Mani and Kumar, 2014). The bioremediation processes rely entirely on natural biological potency. The majority of bioremediation methods depends on several parameters such as soil structure, pH of the polluted sites, moisture content, type of the pollutants, nutrient supplement, microbial diversity, the temperature of treatment sites, and oxygen availability (Atagana et al., 2003; Thapa et al., 2012; Mangunwardoyo et al., 2013; Mani and Kumar, 2014). Bioremediation can occur naturally in a polluted site, which is called natural attenuation.

Lombi and Hamon (2005) have divided bioremediation into ‘*in-situ*’ and ‘*ex-situ*’ strategies. *In-situ* or on-site bioremediation is the most preferred option for removing contaminants from polluted soil and water. In the *in-situ* process, the soils remain confined to their initial location throughout the reclamation process, ending up in minimal site disturbance, fewer public health risks associated with excavation and off-site transport of contaminated soil, and reduced the overall cost over other remediation technologies (Hellekson, 1999; Lombi and Hamon, 2005). The *in-situ* bioremediation is broadly classified into two types, intrinsic and engineered bioremediation (Hazen, 2010). Intrinsic bioremediation takes place through the stimulations of indigenous microorganisms by supplying them with nutrients and oxygen to boost their metabolic activity. This is an unstimulated, unmanipulated, and unenhanced biological remedy of contaminates. Whereas for engineered bioremediation, a specific type of microorganisms or genetically engineered bacteria are introduced into the contaminated place to accelerates the degradation process by creating a conducive physicochemical condition (Kumar et al., 2011).

On the other side, *ex-situ* bioremediation methods require the excavation of polluted soil and water from its original location for the treatment. This is further categorized as a solid-phase system and slurry phase system. Solid-phase bioremediation includes contaminated waste such as industrial waste, domestic waste, municipal solid waste, and sewage sludge with organic waste including manure, leaves, and agricultural waste. The treatment process includes composting, soil biopile, hydroponics, and land farming, which create suitable conditions for indigenous anaerobic and aerobic microorganisms to boost the reclamation process (Kumar et al., 2011; Rayu et al., 2012). From which in hydroponics methods plants are grown in the mineral nutrient solution. Nowadays, this method is a common step for screening the suitable plant for phytoremediation by characterization of its response to heavy metal stress. On the other hand, slurry phase bioremediation is a speedy process where contaminated soils are mixed with additives and water in a bioreactor to create an appropriate environment for microorganisms to eliminate the contaminants.

## Mechanisms of Bioremediation

Both *in-situ* and *ex-situ* remediation methods work on the principle of biotransformation/biodegradation, removal, mobilization, immobilization, or decontamination of various pollutants from the environment through the action of microorganisms (bacteria, fungi, and yeast) and plants (Abatenh et al., 2017). Microbes use chemical contaminants as an energy source during biotransformation and metabolize the target contaminant into useable energy *via* redox reactions. There are usually less harmful by-products or metabolites released back into the environment compared to the primary pollutants. For instance, microorganisms can degrade petroleum hydrocarbons through aerobic respiration in the presence of oxygen. The hydrocarbon gets oxidized by losing electrons, whereas the oxygen reduces by gaining electrons. Water and carbon dioxide are formed as a by-product of this redox reaction (Nester et al., 2001).

**Figure 1:**
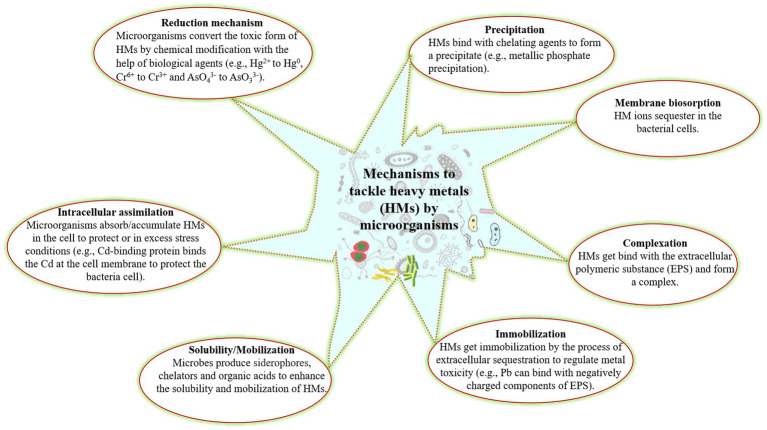
Different microorganisms mechanisms to tackle the HMs from the soil.

The microorganisms play an important role in HM remediation from the contaminated soil as they have acquired various mechanisms to tolerate the toxic effects of HMs. Microorganisms can sequester, precipitate, biosorb, and change the oxidation states of various metals (Ndeddy Aka and Babalola, 2016; Yin et al., 2019; Rizvi et al., 2020; Ibrahim et al., 2021). Metal sequestration happens by cell wall components and by intercellular metal bindings peptides and proteins such as metallothionein, phytochelatins with bacterial siderophores (Ojuederie and Babalola, 2017; Balzano et al., 2020). Microorganisms convert the toxic metal into a less toxic or innocuous form with the help of enzymes (such as dioxygenases, peroxidases, and oxidoreductases). The mechanisms applied by microorganisms to remove HMs from the contaminated soil or convert to less toxic form have been presented in Figure 1. However, the biosorption mechanism is based on two way: first depends on cell metabolism and second on the location of the cell where the HM is removed.

Three key bioremediation ingredients are (1) the presence of a contaminant, (2) the acceptor of electrons, and (3) the existence of microorganisms that can degrade a specific contaminant. Generally, the biodegradation process is easy for the naturally occurring contaminant or those have chemical similarities with naturally occurring compounds. It is due to the potential of microorganisms to destroy the contaminants. For instance, petroleum hydrocarbons are naturally derived chemical products, therefore microorganisms are habituated for these contaminants and can degrade them easily. Different approaches applied in the microbial remediation process such as bioattenuation, biostimulation, bioaugmentation for removing the toxic pollutants from the contaminated land, have been described below.

### Bioattenuation

The contaminants are converted to less harmful or immobilized forms during bioattenuation. Such processes of immobilization and transformation are primarily attributed to microbial biodegradation and biological transformation (Smets and Pritchard, 2003), and, to some degree, to reactions with naturally occurring chemicals and geological media sorption. Contaminant-specific processes of natural attenuation are considered as methods for the remedy of fuel components [e.g., biosparging of benzene, toluene, ethylbenzene, and xylene (BTEX)], but not for other various classes of contaminants (e.g., sulfide and ferrous iron; Atteia and Guillot, 2007).

### Biostimulation

This includes modification in environmental parameters, such as restricting nutrients supplement such as slow-release fertilizers, biosurfactants, and biopolymers (Kumar, 2019), which helps to remove the heavy metal, hydrocarbons and oil contaminants (Junior et al., 2019; Sun et al., 2019, 2021). It also enhances the bioavailability of Cu, Cd, Pb, and Zn, heavy metal uptake, translocation, and biodegradation rate of hydrocarbons, pesticides and herbicides by naturally existing microorganisms present on the site (Lim et al., 2016; Kumar, 2019). There are various fertilizers available as nutrients for microbes to stimulate, e.g., water-soluble NaNO_3_, KNO_3_, NH_3_NO_3_, slow-release customizable, max-bac, IBDU, and oleophilic Inipol EAP22, MM80, F1, S200.

### Bioaugmentation

Bioaugmentation basically increases the heavy metal removal efficiency by introducing the pre-grown microorganisms. In this process, natural/exotic/engineered microbes are incorporated artificially in the heavy metal contaminated soil (Hassan et al., 2019, 2020a). Microbes are collected from the remediation site, separately cultured, genetically grown, and returned to the location. This process helps increase the growth and population of microorganisms, which enhance the solubility, mobility, accumulation of HMs, and increase the remediation efficacy (Atigh et al., 2020). However, it also reduces the risk of these pollutants either through chemically altering their chemical structure or by decreasing their bioavailability (Mandal et al., 2016; Hassan et al., 2019; Zanganeh et al., 2021). Recently this method is applied to various HM contaminated soil using different types of bacteria and fungal strains which include *Oscillatoria* sp., *Leptolyngbya* sp., *Portulaca oleracea, Perenniporia subtephropora, Aspergillus niger* MH541017*, Daldinia starbaeckii, Tremates versicolor*, and *Tremates versicolor* (Atigh et al., 2020; Hassan et al., 2020a; Zanganeh et al., 2021).

## Plant-Based Bioremediation

Plants are used for bioremediation either alone or in combination with microbes (Ramos et al., 2005) instead of depending on microbes and their efficacy in achieving bioremediation of any contaminated medium. The application of green plants to clean up any contaminated medium or surface is not a novel concept. Plants were proposed for treating the wastewater around 300 years ago (Hartman, 1975). Presently a number of plant species such as *Amaranthus spinosus, A. hypochondriacus Chrysopogon zizanioides, Brassica juncea*, *Ricinus communis, Chromolaena odorata, Ageratum conyzoides, Ipomoea carnea, Prosopis juliflora, Lantana camara, Parthenium hysterophorus, Fagopyrum esculentum, Odontarrhena chalcidica, Tagetes patula, T. erecta, and Odontarrhena chalcidica,* have been identified which helpremediate HM contaminated soil (Bauddh and Singh, 2012; Bauddh and Singh, 2015; Huang et al., 2019; Chen et al., 2020a; Raza et al., 2020; Biswal et al., 2021; Cui et al., 2021; Gonzaga et al., 2021; Nugroho et al., 2021; Singh et al., 2021). In addition, plants like *Nicotiana tabacum*, *Arabidopsis thaliana, Beta vulgaris* and *Sedum alfredii* have been genetically modified with suitable bacterial genes from *Caenorhabditis elegans, Saccharomyces cerevisiae, Streptococcus thermophilus, Pseudomonas fuorescens* and employed for remediating the targeted contaminants (Daghan et al., 2013; Liu et al., 2015a; Wang et al., 2019; Nedjimi, 2021). For instance, mercury (Hg) reductase bacterial genes, e.g., merA and merB have been applied in plants for the detoxification of methyl-Hg (Li et al., 2020a). In addition, various biostimulators, such as manure and organic amendments (e.g., various plant biochar, biosolids, and litter) are used in this plant-based bioremediation. Use of different chelators such as citric acid, ethylene diamine tetraacetic acid (EDTA), [S,S]-ethylenediaminedisuccinic acid (EDDS), ethylenediamine-di-o-hydroxyphenylacetic acid (EDDHA), diethylenetriaminepentaacetic acid (DTPA), ethylene glycol tetraeacitic acid (AGTA), nhydroxyethylenediaminetriacetic acid (HEDTA), fulvic acids, salicyclic acid, and tartaric acid control metal sorption, and precipitation through the formation of metal chelate complexes, which consequently enhance the bioavailability of these metals and also improve phytoextraction efficiency (Caporale and Violante, 2016; Acuña et al., 2020; Saleem et al., 2020). The addition of chelates in soils can move more metals into soil solution *via* the suspension of precipitated compounds and desorption of sorbed species. Plants can also naturally produce various phytosiderophores, organic acids, and carboxylates, which can enhance metal mobility, solubility, and bioavailability in soils, thus increasing the phytoremediation potential of plants (Vithanage et al., 2012; Gupta and Singh, 2017). For instance, *Miscanthus sinensis* can detoxify Al by producing various phytosiderophores such as citric acid, malic acid, and chlorogenic acid and stored the metal in cell walls (Haruma et al., 2019).

Plant-based bioremediation is considered a potential tool for the accumulation, transformation, and immobilization of a low level of contaminants (Rayu et al., 2012). The mechanisms behind plants facilitate the reclamation of the polluted soils and groundwater are presented in Table 1. The approach of plant-based bioremediation has several merits such as cost-effectiveness, public acceptance, and the ability to remove inorganic and organic contaminants simultaneously. In a study, mixed mercury-trichloroethylene (Hg-TCE) pollutants are removed by transgenic alfalfa plants pKHCG co-expressing human P450 2E1 (CYP2E1) genes and glutathione S-transferase (GST; Zhang et al., 2013). A major synergistic effect caused by simultaneous expression of CYP2E1 and GST leads to increased accumulation and resistance of heavy metal–organic complex pollutants. Another study by Tammam et al. (2021) found that the plant *Glebionis coronaria* can eliminate Pb from the contaminated soil. It is also recorded that the foliar spray of Indole-3-acetic acid (IAA) and gibberellic acid (GA3) enhanced the growth significantly and increase the phytostabilization capacity of the studied plant. The application of bamboo biochar with the *Salix psammophila* to remediate the multi-metal contaminated soil, enhance the translocation factor (TF) and bioconcentration factors (BCF) of Cd, Cu and Zn (Li et al., 2021a). The higher TF for Zn (TF > 1) and BCF for Cd (BCF > 1) makes *S. psammophila* a potential candidate for the phytoremediation in BBC amendment soil. Recently several studies found that the application of nanoparticles such as Ag nanoparticles (AgNPs), nano-TiO_2_ particles, nanoscale zero-valent iron (nZVI), salicylic acid nanoparticles (SANPs) and magnesium oxide (MgO) nanoparticles along with plants *Zea mays, Glycine max, Isatis cappadocica, Lolium perenne, Boehmeria nivea* and *Raphanus sativus* enhance the growth and phytoextraction of HMs Cd and Pb (Khan and Bano, 2016; Singh and Lee, 2016; Gong et al., 2017; Souri et al., 2017; Huang et al., 2018; Hussain et al., 2019).

**Table 1:** List of various phytoremediation mechanisms and plant species used in various process.

Technique	Mechanism	Plant used	Plant parts	Surface medium	References
Phytoextraction	Uptake and accumulation of heavy metal into plant tissues with subsequent elimination of the plants	*Brassica juncea Amaranthus hypochondriacus, Thlaspi caerulescens*	Roots, Shoot, Leaves	Soils	Odoh et al., 2019; Cui et al., 2021; Singh et al., 2021
Phytodegradation/Rhizodegradation	Enzyme catalysed metabolism by rhizosphere-dwelling microorganisms to transform organic contaminant into simpler molecules	*Rhizophora mangle, Salix viminalis, Vetiveria zizanioides, Typha latifolia*	Roots, Leaves	Surface water, Groundwater	Sampaio et al., 2019; Papadopoulos and Zalidis, 2019; Nedjimi, 2021
Phytostabilization	Decreases the mobility and migration of soil contaminants	*Atriplex undulata, Salix alba, Glebionis coronaria*	Roots	Soils,Groundwater, Mine tailing	Mataruga et al., 2020; Li et al., 2021; Tammam et al., 2021
Rhizofiltration	Uptake of metals *via* plant roots	*Eichhornia crassipes, Lemna minor, Pistia stratiotes*	Roots	Surface water, Water pumped	Kodituwakku and Yatawara, 2020; Singh et al., 2021
Phytovolatilization	Removal of pollutants such as selenium, mercury, volatile hydrocarbons *via* evapotranspiration processes	*Arundo donax, Stanleya pinnata, Brassica juncea, B. Napus*	Roots, Leaves	Soils, Groundwater	Guarino et al., 2020; Hasanuzzaman et al., 2020; Yan et al., 2021
Phytostimulation	Phytostimulation (a symbiotic relationship that exists between plants and several soil microorganisms) is developed for the remediation of polychlorinated biphenyl (PCBs)	*Brassica campestris, Zea mays, glycine max*	Roots	Soils	Zahoor et al., 2017; Bilal et al., 2020

Plants are effective in extracting inorganic and organic pollutants from the ground through the roots, they can also be transported and accumulated (phytoextraction/accumulation) in the harvestable parts of the plant (Pranaw et al., 2020). Transpiration to the atmosphere *via* leaf stomata (phytovolatilization) occurs in some instances (Rascio and Navari-Izzo, 2011). Phytodegradation of organic compounds are metabolized by plants in three sequential steps (namely transformation, conjugation, compartmentalization, respectively) with the aid of enzymes, e.g., cytochrome (CY) P450 and GT–glycosyltransferase (GT), which results in the storage of contaminant in the vacuole, incorporation into the cell wall, or excretion from the cell. In addition, plant-associated microorganisms in the rhizosphere (rhizodegradation) can degrade organic contaminants (Truu et al., 2015). By releasing root exudates and other compounds (e.g., organic acids) to the surrounding soil along with providing a surface for microbe colonization, plants can promote the biodegradation of pollutants, thereby contributing to the increased density and metabolic activity of microorganisms (rhizosphere effect) and contaminant bioavailability. Plant supplements nutrients to endophytic bacteria and stimulates catabolic gene expression. In turn, endophytic bacteria degrade organic contaminants, thereby reducing phytotoxicity and producing hormones (Shukla et al., 2020).

Since metal bioavailability in soils is relatively poor under most conditions, plants have very active metal uptake systems that utilize transporter molecules such as Zn-regulated transporter protein, Cu transporter protein, etc. (Krämer et al., 2007). In addition, plants are capable of acidifying the soil and mobilize soil-bound metals by secreting metal-chelating molecules to the surrounding soil, such as siderophores (catechol and hydroxymate), organic acids (e.g., citrate and malate), biosurfactants (rhamnolipids), protons from the root exudates (Yan et al., 2020; Bruno et al., 2021). Heavy metals cannot be biodegraded inside the plant, unlike organic contaminants, but can only be converted from one oxidation state/organic complex to another. It ends up in metal accumulation inside the plant. There are nearly 450 hyperaccumulator plants varies from annual to perennial herbs, shrubs, and trees (e.g., *Brassica juncea, Zea mays, Ricinus communis, nicotiana tabacum, Helianthus annuus, Pteris vittata, Thlaspi caerulescens, Russian thistle, Sesbania drummondii, Salix matsudana, Populus deltoides*), which have been identified to accumulate, metabolize and depollute extraordinary high concentration of metal ions (such as Cd, Pb, Ni, Co, Mn, Zn) in their above-ground tissues (Meagher, 2000; Padmavathiamma and Li, 2007; Shah and Nongkynrih, 2007; Sheoran et al., 2009; Palanivel et al., 2020).

## Microorganism-Based Bioremediation

**Figure 2:**
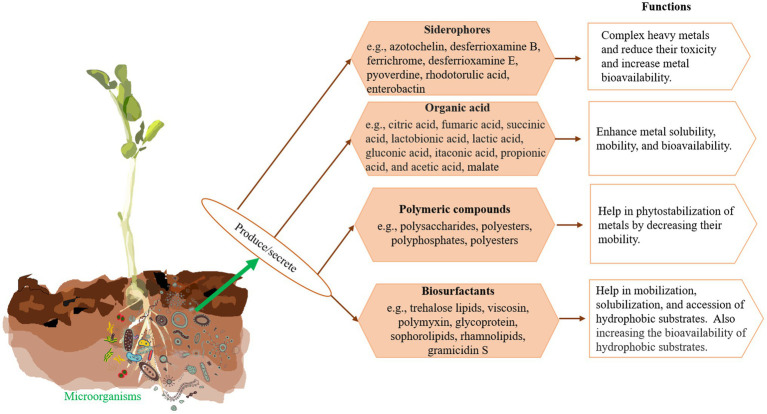
Microorganisms produce/secrete different compounds and their role in bioremediation.

The capacity of microorganisms to degrade contaminants depends on their metabolic system through which the pollutants alter to innocuous form *via* the redox process (Jan et al., 2014). They help plants alleviate metal toxicity by sequestration of metals in cell wall components, alteration of the biochemical pathway to block metal uptake, reduction of the intercellular metal concentration *via* a precise efflux system, and conversion of poisonous metals to a less harmful state (Jan et al., 2014; Ojuederie and Babalola, 2017). Microorganisms (such as bacteria and fungi) play a vital role in the microbial bioremediation process. In addition, microorganisms contain several genes located in transposons and plasmids, which encode heavy metal resistant proteins and transporters. Recently, Kang et al. (2016) found that four bacterial strains, namely *Enterobacter cloacae* KJ-46*, E. cloacae* KJ-47, *Sporosarcina soli* B-22, and *Viridibacillus arenosi* B-21 had synergistic effects on the remediation of Cd, Pb, and Cu from contaminated soil. Moreover, the combination of bacteria strains shows greater resistance and efficacy for metal bioremediation compared to a single strain after 48 h of experiments. Microbes secrete several metabolites that play a significant role in bioremediation of contaminated sites (Sobrinho et al., 2013; Dixit et al., 2015; Coelho et al., 2015; Ahemad, 2019; Figure 2).

Bacteria generate siderophores that can diminish metal bioavailability and are subsequently eliminated from contaminated land (Ahemad, 2019). It is recorded that bacterial cell can alter their morphology to increase the production of siderophores for promoting the intercellular accumulation of metals (Manoj et al., 2020). Chibuike and Obiora (2014) found that a sulfate-reducing bacterium *Desulfovibrio desulfuricans* can alter sulfate to hydrogen sulfate, which further reacts with HMs (Cd and Zn) and then form insoluble metal sulfides. The biomolecules of microbial cell walls contain negatively charged functional groups such as phosphate, hydroxyl, and carbonyl, which bind quickly with toxic metal ions and help them in bioremediation (Coelho et al., 2015; Dixit et al., 2015). Besides, bacteria can be grown and survive in any control and intense environmental conditions, making them a perfect bioremediation agent (Srivastava et al., 2015).

Likewise, fungi can be grown in harsh environmental conditions and detoxify metal ions by accumulation, valence transformation, and extra and intracellular precipitations (Ayangbenro and Babalola, 2017). In addition, fungi act as a promising biocatalyst in the bioremediation process, where they absorb toxic chemicals into their spores and mycelium. Recently, Hassan et al. (2020a) showed the bioremediation capability of fungal consortia of *Ascomycota* and *Basidiomycota*, suggesting fungal bioaugmentation helps decontaminate heavy metal from contaminated land. A number of investigations are carried to study the microorganism bioaccumulation and biosorption capacity for effectively remediate metal-contaminated environment (Table 2).

**Table 2:** Different microorganisms and their bioaccumulation and biosorption capacity.

Microorganism(s)	Contaminant(s)	Remarks	References
*Scenedesmus acutus, Chlorella pyrenoidosa*	Cd	*C. pyrenoidosa* and *S. acutus* accumulated 3 and 1.5% of Cd and biosorbed 97 and 98.5% of Cd, respectively.	Chandra et al., 2020
*Aspergillus* spp.	Cd, Cu	The removal efficiency for Cu and Cd was recorded >90%. The biosorption potential of living and dead cells for Cd was 0.1977 and 0.1772 mg g^−1^ and for Cu it was 5.3676 and 18.661 mg g^−1^, respectively.	Hasgül et al., 2019
*Streptomyces* K11	Zn	The bioaccumulation capacity was 4.4 mmol g^−1^. The maximum biosorption capacity recorded was 0.75 mmol g^−1^.	Sedlakova-Kadukova et al., 2019
*Bacillus xiamenensis* PbRPSD202	Pb, Cd, Cr, As, Ni, Cu, and Zn	The maximum Pb biosorption capacity for living and dead biomass of *B. xiamenensis* shows 216.75 and 207.4 mg g^−1^, respectively.	Mohapatra et al., 2019
*Aspergillus flavus* SFL	Cr	The intercellular accumulation of *A. flavus* SFL was 50% more than the reference strain.	Vajpai et al., 2020
*Phanerochaete chrysosporium*	Cd^+2^, Ni^+2^	The accumulation efficiency of *P. chrysosporium* for Cd^2+^ and Ni^2+^ was 96.23 and 89.48%. The maximum biosorption capacity for Cd^+2^ and Ni^+2^ recorded 71.43 and 46.50 mg g^−1^, respectively.	Noormohamadi et al., 2019
*Pseudomonas azotoformans* JAW1	Cd, Pb, and Cu	Metal accumulation occurs on the cell surface (biosorption). The maximum adsorption found of Cd, Pb, and Cu by 98.57, 88.57 and 69.76%, respectively. The removal level achieved the highest in order of Pb (78.23%), Cu (63.32%), and Cd (44.67%).	Choińska-Pulit et al., 2018
*Aspergillus tamari, Simplicillium subtropicum, Aspergillus niger, Fusarium solani,*	Cu	Although *A. tamari* and *S. subtropicum* growth rate was low, the intake of Cu per unit of biomass is high compare to two other species.	Ong et al., 2017
*Ensifer adhaerens* OS3	Cd, Cr, Ni, Pb, Cu, and Zn	The maximum accumulation was recorded for Ni (95%) and lowest for Pb (74%) and in order of Ni > Cu > Zn > Cr > Cd > Pb. Biosorption capacity recorded in order of Zn > Cr > Cd > Ni > Cu > Pb.	Oves et al., 2017

Recently researchers have been isolated various heavy metal resistance microorganism from contaminated lands, mining dumping and abandoned sites, industrial waste dumping yards, and the rhizosphere of plants growing in metal-contaminated sites (Banerjee et al., 2019; Aguilar et al., 2020; Akhter et al., 2020; Nurfitriani et al., 2020; Din et al., 2021; Sharma and Shukla, 2021b). The isolated bacterial genera (such as *Arthrobacter, Enterobacter, Corynebacterium, Stenotrophomonas, Bacillus*, and *Pseudomonas*) and fungi (such as *Aspergillus flavus, Aspegillus awamori, Saccharomyces cerevisiae, Phanerochaete chrysosporium, Penicillium oxalicum*, and *Trichoderma viride*) play a significant role in bioremediation process. Bacteria and fungi precisely used to eliminate the specific metals in recent years have been reviewed and presented in Tables 3, 4, respectively.

**Table 3:** Metal bioremediation potential of bacteria strains.

Targeted heavy metal	Bacteria used	Remarks	References
Cd and Pb	*Enterobacter cloacae, Klebsiella edwardsii* and *Pseudomonas aeruginosa*	*P. aeruginosa* showed the highest bioremediation potential compared to the other two with 58.80 and 33.67% of remediation in 50 mg Cd L^−1^ and 300 mg Pb L^−1^, respectively.	Oziegbe et al., 2021
Pb and Ni	*Ochrobactrum intermedium* BPS-20 and *Ochrobactrum ciceri* BPS-26	*O. intermedium* BPS-20 and *O. ciceri* BPS-26 accumulated Pb by 85.34 and 71.20% and Ni by 74.87 and 88.48%, respectively.	Sharma and Shukla, 2021a
Pb	*Bacillus cereus BPS-9*	BPS-9 strains recorded the highest Pb accumulation potential of 79.26% and the biosorption capacity was 193.93 mg g^−1^.	Sharma and Shukla, 2021b
Cr, Pb, and Ni	*Klebsiella pneumoniae* MB361, *Stenotrophomonas* sp. MB339, and *Staphylococcus* sp. MB371	The percentage of accumulation increase gradually with time and increased biomass. The highest removal was recorded by MB339 with Pb (85.30%), and Ni (48.78%), followed by MB361 with Cr (83.51%), while MB371 sorbed Pb by 88.33%.	Aslam et al., 2020
Ni	*Pseudomonas* sp. P21, *Stenotrophomonas* sp. S20, and *Sphingobium* sp. S42	Bacterial strains S20 and P21 show high tolerant levels to Ni up to 400 mg L^−1^, while S42 removed 33.7% of metal.	Chen et al., 2020
Hg	*Fictibacillus nanhainensis* SKT-B and *Bacillus toyonensis* PJM-F1	*F. nanhainensis* SKT-B accumulated the highest level of Hg followed by *B. toyonensis* PJM-F1 with 82.25 and 81.21%, respectively.	Nurfitriani et al., 2020
Co and Ni	*Anoxybacillus mongoliensis*	The highest accumulation by bacteria recorded for Co and Ni was 274.9 and 268.5 mg g^−1^, respectively. Further, increasing activities of superoxide dismutase (SOD) and catalase (CAT) were also recorded.	Akkoyun et al., 2020a
Pb, Cd, and Ni	*Rhizopus stolonifer* and *Bacillus megaterium*	When growing the bacteria separately, *R. stolonifer* and *B. megaterium* recorded maximum uptake of Cd and Ni by 479.10 and 501.05 mg L^−1^, respectively. Overall *B. megaterium* uptake a higher concentration of combined HMs.	Njoku et al., 2020
Cr	*Bacillus cereus* AVP12 and *Bacillus cereus* NC7401	The highest Cr accumulation potential of AVP12 and NC7401 strains isolated from the contaminated sites was 181.0 and 107.5 mg L^−1^, respectively. While for the same strains AVP12 and NC7401 isolated from non-polluted sites were 92.59 and 62.11 mg L^−1^, respectively.	Akhter et al., 2020
Hg and Pb	*Exiguobacterium profundum*	The highest bioaccumulation of Pb and Hg for *E. profundum* were 54.35 and 37.56 mg g^−1^, respectively.	Akkoyun et al., 2020b
Cr, Ni, and Pb	*Lactobacillus plantarum* MF042018	It shows high tolerance against the Ni and Cr up to 500 and 100 ppm, respectively. The biosorption capacity of MF042018 was recorded very high for Cd and Pb at pH 2.0 and temperature 22°C after 1 h.	Ameen et al., 2020
As	*Bacillus cereus* and *Lysinibacillus boronitolerans*	The bacterial strains P2IIB, P1C1Ib and P2Ic resistant to 3,000 mg L^−1^ of As. The bacteria culture removes 85.72% of arsenate and 71.88% of arsenite from the medium.	Aguilar et al., 2020
Cr	*Bacillus cereus*	The bacteria strain can tolerate Cr_2000_ (2,000 mg L^−1^) Cr(VI) and can completely decrease Cr_200_ under heterotrophic conditions within 16 h. It is recorded that Cr(VI) was effectively reduced to Cr(III).	Banerjee et al., 2019
As	*Ochrobactrum ciceri* SW1 and *Exiguobacterium profundum* PT2	Both bacterial strains increased production of EPS in the presence of As, which help to sequester arsenic.	Saba et al., 2019
Hg, Cd, Pb, Cu, Ni, and Zn	*Escherichia coli* K-12	The bacterial strain can absorb different types of metal ions. It can absorb more than 30 varieties of metal ions *via* its outer membrane.	Jin et al., 2018
Cd	*Cupriavidus necator* GX_5, *Sphingomonas sp.* GX_15, and *Curtobacterium* sp. GX_31	The highest removal capacity of Cd recorded in order of GX_31, GX_15 and GX_5 with 86.06, 53.88 and 25.05%, respectively.	Li et al., 2018

**Table 4:** Metal bioremediation potential of fungi strains.

Target heavy metal	Fungi used	Remarks	References
Cd	*Penicillium chrysogenum* FMS2	The highest tolerance level recorded for *P. chrysogenum* FMS2 was 1,000 mg L^−1^. The fungal strain can survive in the wide environmental condition such as temperature and pH range between 15–35°C and 4.0–12.0, respectively. The Cd removal capacity of fungi was approximately 49% in 15 days of exposure.	Din et al., 2021
Cd, Cu, Ni, Pb, and Zn	*Ganoderma lucidum*	The concentration of Pb, Zn, Ni, Cu and Cd in contaminated soil were 4,490, 147, 27.7, 19.4 and 2.18 mg kg^−1^ and *G. lucidum* accumulated 138, 29.8, 3.48, 3.69 and 1.01 mg kg^−1^ of respective metal after inoculated in contaminated soil.	Ipeaiyeda et al., 2020
Pb	*Aspergillus niger, Penicillium oxalicum*, and *Trichoderma asperellum*	*Trichoderma, Penicillium* and *Aspergillus* accumulate Pb ions by 75.29, 66.77, and 56.82%, respectively.	Mariconi et al., 2020
Pb, Ni, and Zn	All isolated fungi, *Ascomycota* and *Basidiomycota*	The highest bioremoval capacity for Ni and Pb was 52 and 44% from the bioaugmented soil with all isolated fungi. While for Zn, the maximum removal was 36% in *A. consortium*-treated soil. Overall, Pb and Ni removal efficacy in order of isolated fungi > *Basidiomycota > Ascomycota*, whereas for Zn it was *Basidiomycota* > all isolated fungi > *Ascomycota*.	Hassan et al., 2020a
As, Cr, Cu, Mn, and Fe	All isolated fungi, *Ascomycota* and *Basidiomycota*	Fungal consortia show the highest tolerance index of 1.0 for Cr, Cu and Fe in agar medium. Further, the consortium of all isolated fungi shows the removal capacity of As, Mn, Cr, and Cu by 77,71, 60 and 52%, respectively.	Hassan et al., 2020b
As	21 fungal strains including *Humicola* sp.	All the isolated fungal strains can tolerate up to 5,000 mg L^−1^ AsV. The accumulation capacity of fungi biomass ranged between 0.146 to 11.36 g kg^−1^ and volatilization of As between 0.05 to 53.39 mg kg^−1^ biomass. *Humicola* sp. recorded the highest biovolatilization capacity by 53.39 mg kg^−1^.	Tripathi et al., 2020
Hg	*Penicillium spp.* DC-F11	DC-F11 fungal strain detoxified Hg *via* extracellular sequestration through precipitation and adsorption.	Chang et al., 2020
Hg	*Aspergillus* sp. A31, *Lindgomycetaceae* P87*, Curvularia geniculata* P1, and *Westerdykella* sp. P71	All four species of endophytic fungi remove up to 100% of Hg in a species-dependent manner from the culture medium.	Pietro-Souza et al., 2020
Cd	*Aspergillus fumigatus*	*A. fumigatus* showed the highest tolerance against Cd with a removal percentage of 74.76 and uptake capacity of approximately 5.02 mg gm^−1^.	Talukdar et al., 2020
Cd and Pb	*Simplicillium chinense* QD10	Cd biosorption occurs with forming Cd-chelate and Pb mainly adsorbed by extracellular polymeric substances (EPA).	Jin et al., 2019
Cu, Cd, Pb, and Zn	*Alternaria chlamydosporigena, Trichoderma harzianum, Acremonium persicinum, Fusarium verticillioides, Seimatosporium pistaciae,* and *Penicillium simplicissimum*	*T. harzianum* was found the maximum tolerant against Cd, Cu and Pb. *A. persicinum* and *P. simplicissimum* record the highest biosorption and accumulation of HMs.	Mohammadian et al., 2017
Cd, Cr, Cu, Ni, and Zn	*Beauveria bassiana*	It removed 84% multi-metal from the mixture sample while individual metal removal capacity was 61–75%. *B. bassiana* removed the metal *via* accumulation and sorption processes.	Gola et al., 2016
Cu, Pb	*Aspergillus flavus* and *A. niger*	The biosorption of Cu and Pb by *A. flavus* and *A. niger* was recorded 81.8 and 83.1%, respectively, during the initial 10 min.	Iram et al., 2015

## Plant–Microbe Associated Remediation

The microorganism-plant-based remediation has gain popularity currently due to its higher removal efficiency compared to plant-based remediation process. These microorganisms are involved in the various biochemical process such as carbon and nitrogen mineralization, nitrogen fixation, and decomposing organic matter, which contributes to soil formation, nutrient cycling and transfer of energy. Soil microorganisms are also affected by HMs in contaminated areas. However, with continuous exposer, they tend to tolerate and develop unique features with few specific microbial populations. These types of specific microbes can be employed for remediating toxic metals from contaminated lands. Further, soil microorganisms that form a symbiotic association with host plants are the most successful species in the soil reclamation process. The mycorrhizal fungi form intimate symbiotic relationship with host plants, which have been applied in many bioremediation processes (Yang et al., 2015; Gunathilakae et al., 2018; González-Chávez et al., 2019; Rubin and Görres, 2021). The arbuscular mycorrhizae as the most well-known symbiotic fungi are frequently used in phytoremediation due to their ubiquity in soil. They can develop several mechanisms to tolerate high metal concentrations in soils, thus promoting plant growth (Janoušková et al., 2005; Fasani et al., 2018). In addition, plant growth-promoting bacteria (PGPB) can also stimulate plant growth activities and help plants cope with the contaminated ecosystem. They can enhance plant growth through direct and indirect mechanisms that are discussed in the separated section below.

There are two aspects of plant–microbe-based bioremediation process. First of all is the microorganisms help the host plant sustain in the harsh environmental condition by providing nutrients. Second, the plant plays a critical role by maintaining favorable environmental conditions such as improving soil organic matter, available P, K, and N, where soil microorganisms can thrive and enhance the reclamation process. Recently, a number of studies have been highlighted both side benefits of the plant–microbe-based bioremediation process. A study recorded that planting of *Trifolium repens* in heavy metal contaminated sites improves soil enzymatic activities (Lin et al., 2021). Wang et al. (2021) also showed that plantation of *Salix* in Cd contaminated soil increased beneficial microorganisms diversity, such as genera of bacteria include *Arthrobacter, Bacillus, Flavobacterium, Niastella, Novosphingobium, Niabella*, *Anaeromyxobacter, Rmlibacter, Solitalea, Devosia, Mesorhizobium Nitrospira, Thermomonas, Flavisolibacter, Pedomicrobium, Lysobacter, Rubrivivax Phyllobacterium*, and mycorrhizal genera of fungi include *Actinomucor, Conocytes, Amanita, Cryptococcus, Xylaria, Ramicandelaber, Spizellomyces, Sporobolomyces, Rhodotorula Umbilicaria, Claroideoglomus, Tilletiopsis,* and *Cirrenalia* in plant rhizosphere.

### Plant Growth-Promoting Bacteria

It is well known that PGPB can enhance phytoremediation efficiency (Ma et al., 2016; Lin et al., 2021; Kumar et al., 2021a). The PGPB may directly prompt root proliferation and improve plant growth and fitness, plant metal resistance, uptake and translocation of nutrients and metals, and protect plants from phytopathogens (Ma et al., 2011; Gupta et al., 2013; Fasani et al., 2018) by producing and secreting various organic acids, polymeric compounds, chelators, and hormones such as indole-3-acetic acid (IAA), 1-aminocyclopropane-1- carboxylate (ACC) deaminase, polysaccharides, glomalinand, azotobactin, azotochelin, alcaligin E, pyochelin, coelichelin, ferrioxamine B, and pyoverdin, which are responsible for decrease the soil pH and enhance the metal bioavailability, whereas the polymeric compounds help in phytostabilization of metals by decreasing their mobility (Chen et al., 2017). The chelators work as metal-binding ligands to enhance metal bioavailability, improve root-shoot translocation and metal uptake capacity, and facilitate intracellular heavy metal accumulation in organelles (Yan et al., 2020). Inoculation of ACC deaminase-producing PGPB showed extensive root and shoot density along with increased biomass and phytoremediation efficiencies (Arshad et al., 2007; Yan et al., 2020). It is found that *Bacillus* sp. XZM lowers As toxicity to the plant by producing a higher amount of extracellular polymeric substance (EPS), siderophore, and IAA (Irshad et al., 2020). Some of PGPB, such as *Pseudomonas, Micrococcus, Erwinia, Azospirillium, Flavobacterium, Azotobacter, Chromobacterium*, and *Agrobacterium* have been applied in the phytoremediation process (Bhattacharyya and Jha, 2012; Ma et al., 2019). Ma et al. (2016) isolated two droughts resistant serpentine PGPB *Pseudomonas reactans* Ph3R3 and *Pseudomonas libanensis* that showed high resistance to different HMs (Cd, Cr, Pb, Cu, Ni, and Zn), salinity, extreme temperature, and antibiotics. Both strains significantly enhanced plant growth, pigment content, and leaf relative water, and also translocation and bioconcentration factors for Cu and Zn under the drought condition.

Further, PGPB are found to be an important player in remediating the HM contaminated marine ecosystems. The study by Mesa-Marín et al. (2020) recorded that inoculation of *Thalassospira australica* SRT8, *Vibrio neocaledonicus* SRT1 and *Pseudarthrobacter oxydans* SRT15, with *Salicornia ramosissima* improved the relative plant growth rate and the number of new branches by 32 and 61%, respectively, when planted in the HM contaminated estuarine soil. The inoculation of PGPB also helps to accumulate the highest concentration of HMs like As, Cd, Cu, Ni, Pb and Zn in the root and subsequently enhance the phytoremediation potential of *S. ramosissima*. In one another study inoculation of *Bacillus flexus* KLBMP 4941 with coastal halophytes *Limonium sinense* under the salt stress ecosystem shows positive effects on the hostplant survival and growth and it can be employed for phytoremediation of saline soils (Xiong et al., 2020). Two PGPB namely *Bacillus cereus* strain P2 and *Planomicrobium chinense* strain P1 isolated by Khan et al. (2018) and inoculated with *Helianthus annus* for phytoremediation of HMs in drought conditions found a significantly positive result. The study confirmed that the application of PGPB and salicylic acid significantly increased the rhizosphere accumulation of Cd, Pb, Ni by 84, 66 and 65%, respectively. In addition, inoculation of PGPB significantly enhanced the root length, shoot length, root fresh, and dry weight by 68, 60, 61, and 63%, respectively. Likewise, in various studies different types of PGPB such as *Bacillus subtilis, Bacillus thuringiensis, Ensifer meliloti* RhOL6 and RhOL8*, Bacillus megaterium, Pseudomonas sp.* DSP17 and *Proteus sp.* DSP1 have been applied along with organic and inorganic amendments found enhanced remediation of HMs from different types of soils which include sandy soil, arid and semi-arid soils (Khan and Bano, 2018; Raklami et al., 2019; Khodaverdiloo et al., 2020).

Generally, associations of leguminous plants with PGPB have also been applied in the phytoremediation process of highly metal-contaminated sites (Hao et al., 2014). But recently, this remediation method is used in less or moderately metal-contaminated agriculture soil (Saadani et al., 2019). Recently, Saadani et al. (2019) found that the inoculation of PGPB with *Sulla coronaria* and *Vicia faba* L. var. *minor* showed a higher metal accumulation in legumes grown in low contaminated agriculture soil compared to non-inoculated legumes. After the cultivation of symbiotic legumes, soil fertility is positively affected with higher organic content (phosphorous and nitrogen) and soil decomposition rate. The rhizobium-legume symbiosis relationship between high metal-resistant *Sinorhizobium meliloti* CCNWSX0020 and plant *Medicago lupulina* has been successfully used in the study for efficient bioremediation of HMs (Lu et al., 2017). It is also recorded that the bacterial strain’s extracellular polymeric substances help to immobilize Cu^2+^. The genetically engineered rhizobium-legume symbiont is also used to remediate the As contamination from the soil. A study by Zhang et al. (2017) inserted the arsenite [As (III)] S-adenosylmethionine methyltransferase gene (*Crars*M) derived from alga *Chlamydomonas reinhardtii* in *Rhizobium leguminosarum* bv*. trifolii* strain R3 and check the As methylation capacity by symbiosis with red clover found a positive result in the test. Likewise, Tsyganov et al. (2020), applied two transgenic strains of *Rhizobium leguminosarum* bv. *viciae*, 3,841-PsMT2 and 3,841-PsMT1 to pea plants (*Pisum sativum*) for the study of Cd tolerance and accumulation in plants. The study concludes that the pair of legume-rhizobia may be applied for phytostabilization purposes.

### Arbuscular Mycorrhizal Fungi

AMF are mostly found in terrestrial plant roots by forming the symbiotic association. In the root cortex, the fungus colonizes and develops a thick extended mycelium around the roots, which acts as an intermediatory connection between plants and soils and helps absorb nutrients from soils (Kernaghan, 2005; Reinhardt, 2007). AMF are also found in highly disturbed ecosystems or polluted soils (Cornejo et al., 2008; Yan et al., 2020). AMF can confer plant metal resistance (Singh, 2012; Xu et al., 2012; Curaqueo et al., 2014; Gunathilakae et al., 2018). AMF is a tremendous biological interest due to its positive effects on symbiotic relationships and remediation capability. Further, it has been exploring in every way to employ AMF for stabilizing the metals in contaminated land. The mycorrhizal plants enhance metal phytostabilization by metal sequestration in roots and hyphae. The metals confined to soils make them less bioavailable. Thus, the toxic effects of metals on other living microorganisms are alleviated.

Many studies have been conducted to investigate the role of AMF in phytoremediation (Table 5). Liu et al. (2015b) conducted a study on Cd uptake capacity of *Solanum nigrum* inoculated with *Glomus versiforme* BGC GD01C (Gv) in different Cd concentrations soil. They found that the inoculation of *G. versiforme* highly improved the total Cd uptake in plants at different Cd concentrations. Many researchers have attempted to explore more possibilities to remediate the contaminants from the stressed environment. Recently, a study conducted by Hao et al. (2021) showed that the phytoremediation potential of *Zea mays* inoculated with *Claroideoglomus etunicatum* grown in Lanthanum (La) contaminated soils enhanced bacterial diversity including *Agrococcus, Lysobacter, Planomicrobium, Microbacterium, Streptomyces, Saccharothrix, Penicillium*, and other unclassified bacteria and fungi like *Penicillium.* This study confirmed that AMF can regulate the rhizosphere fungal and bacterial diversity to foster beneficial microorganisms that help the plant sustain. Further, an investigation is undertaken in Ni contaminated saline soil for remediation using *Helianthus annuus* inoculated with plant beneficial bacteria (*Pseudomonas libanensis* TR1) and AMF (*Claroideoglomus claroideum* BEG210; Ma et al., 2019). The study found that the bacteria and fungi alone or in combination, significantly increase plant growth, physiological parameters, and accumulation of Ni and Na^+^, thus contributing significantly to Ni Phytostabilization, Na^+^ and Ni detoxification, and Na^+^ exclusion. Therefore, bioaugmentation with PGPB with AMF can be used as a useful strategy for reclaiming metal-contaminated saline soil.

**Table 5:** Role of microorganisms in the removal of heavy metals by plants.

Targeted heavy metal	Microorganisms used	Host plant	Remarks	References
Bacteria				
Cd, Cu, Ni, Pb, and Zn	*Bacillus cereus* TCU11	*Zea mays*	TCU11 significantly enhanced the biomass, chlorophyll, carotenoids, proline, phenolics, protein and antioxidant enzymes. It also increased the translocation of metals except for Ni. Overall, it improves the phytoremediation efficiency.	Bruno et al., 2021
Cu	*Pseudomonas lurida* EOO26	*Helianthus annuus*	Inoculation of EOO26 increased the Cu accumulation in roots and leaves by 8.6 and 1.9-fold, respectively, and total plant uptake by 2.6-fold compared to the uninoculated plants.	Kumar et al., 2021a
Cd, Pb, and Cr	*Adhaeribacter, Kaistobacter, Lysobacter, Pontibacter, Flavisolibacter, Bacillus*	*Trifolium repens*	*Kaistobacter, Lysobacter* and *Pontibacter* significantly helped in metal accumulation, whereas the other three species enhanced plant growth.	Lin et al., 2021
Cd	*Micrococcus* sp., *Arthrobacter* sp.	*Chlorophytum amaniense, C. comosum*	*Micrococcus* sp. increased the production of biomass of both plants. Both the bacterial strains boost phytoextraction of Cd.	Sangsuwan and Prapagdee, 2021
Cu	*Pseudomonas* sp. TR15a, *Bacillus aerophilus* TR15c	*Helianthus annuus*	The consortium of bacteria significantly increased the dry biomass, germination, root and shoot Cu accumulation by 64¸ 32, 47 and 75%, respectively.	Kumar et al., 2021b
Cu, Cd, Pb, and Zn	*Bacillus* subtilis, *Bacillus licheniformis -* BC *Streptomyces pactum* Act12 -ACT	*Brassica juncea*	Co-inoculation of bacteria increased the enzyme activity, metal bioavailability, plant growth and phytoextraction capacity of *B. juncea.*	Jeyasundar et al., 2021
Cd	*Lelliottia jeotgali* MR2, *Klebsiella michiganensis* TS8	*Miscanthus floridulus*	Strain TS8 enhanced plant growth and declines the total Cd in the rhizosphere, while MR2 significantly increased the translocation of Cd from root to shoot parts.	Liu et al., 2021a
Cu, Cd, Pb, and Zn	*Bacillus cereus* MG257494.1*, Alcaligenes faecalis* MG966440.1 *Alcaligenes faecalis* MG257493.1	*Sorghum vulgare*	The bacteria consortium increased the microbial activity and reduced metal bioaccumulation in the plant and its root. It also controlled the metals bioaccumulation factor (BAF) in plants and the rhizosphere.	Abou-Aly et al., 2021
Cd, Pb, and Cr	*Pseudomonas putida* RE02	*Trifolium repens*	The inoculation RE02 improved the seed germination tailing, soil fertility and the uptake of total heavy metal by 30.03–574.58%.	Liu et al., 2021b
Cd and Mn	*Enterobacter* sp. FM-1	*Polygonum lapathifolium* L*., Polygonum hydropiper* L.	Inoculation of bacteria increased soil bioavailability of Cd and Mn significantly and lowered the soil pH, resulting in an increase in metal accumulation in both the plants.	Li et al., 2020
Sb	*Pseudomonas fuorescens*	*Trifolium repens*	The application PGPB with nZVI significantly enhanced Sb accumulation capacity of *T. repens.*	Zand et al., 2020
As	*Cupriavidus basilensis* r507	*Pteris vittate*	*P. vittata* accumulated up to 171% of As, when inoculated with the bacterial strain.	Yang et al., 2020
Pb	*Micrococcus luteus*	*Chromolaena odorata*	*M. luteus* inoculated with *C. odorata* can be applied to remediate the moderately Pb-fuel oil contaminated mild saline soil.	Jampasri et al., 2020
As	*Bacillus* sp*. XZM*	*Vallisneria denseserrulata*	The symbiosis between the plant and bacteria significantly enhanced As uptake and removal capacity. In addition, 85% arsenic found as As (III) and > 77% stored in vacuole of leaves cells.	Irshad et al., 2020
Al	*Chaetomium cupreum*	*Miscanthus sinensis*	The bacteria produced siderophore called oosporein that supports seedling growth and increased Al tolerance and accumulation.	Haruma et al., 2019
As	*Azospirillum brasilense* Az39, *Bradyrhizobium japonicum* E109	*Glycine max*	The mortality of plants reduced with an increase in plant growth, nodule number and nitrogen content. As translocation to aerial parts also decreased, thus it enhances the phytostabilization potential of *G. max.*	Armendariz et al., 2019
Cd, Pb Cr, Cu, and Zn	*Mesorhizobium loti* HZ76, *Ensifer adhaerens* HZ14, *Rhizobium radiobacter* HZ6	*Robinia pseudoacacia*	Treatment with *M. loti* HZ76 results in significantly increased nodule number. Overall, the addition of bacteria strains enhanced the phytoremediation efficiency.	Fan et al., 2018
Cd, Pb, and Zn	*Streptomyces* sp. Strain B1, B2, B3	*Salix dasyclados* L.	Bioaugmentation with bacteria significantly enhanced plant biomass and decreased oxidative stress. B1 strain record the high potential for phytoextraction due to its highest ability for siderophore secretion.	Złoch et al., 2017
Pb and U	*Enterobacter sp.* HU38*, Pantoea stewartii* ASI11, *Microbacterium arborescens* HU33	*Leptochloa fusca*	The bacterial consortia increased metal accumulation capacity by 58–97% and 53–88% for Pb and U, respectively.	Ahsan et al., 2017
Fungi				
Cd and Zn	*Rhizophagus irregularis* (FR717169)	*Phragmites australis*	Under Zn stress, the fungi helped increase the activities of ascorbate peroxidase (APX) and SOD. Under Cd stress, CAT, peroxidase (POD), SOD and APX increased significantly. The translocation factor of Zn and Cd reduced by 10–57 and 17–40%, respectively.	You et al., 2021
Cd	*Funnelliformis mosseae*	*Solanum nigrum, Oryza sativa*	Intercropping with fungi enhanced growth and Cd accumulation of *S. nigrum.* The treatments help reduce the Cd level in rice parts with a maximum reduction in brown rice by 64.5%.	Yang et al., 2021
Cd	*Blastocladiomycota, Chytridiomycota, Mortiriellomycota, Tilletiopsis, Sporobolomyces, Cryptococcus, Conocytes,Umbilicaria, Amanita, Xylaria, Cirrenalia*	*Salix*	The presence of fungi showed a positive correlation with Cd accumulation. The study recorded that a higher fungal number contributes to high biomass.	Wang et al., 2021
La	*Claroideoglomus etunicatum*	*Zea mays*	The AMF promoted nutrient uptake and growth of *Z. mays* in various La stressed soil. It also increased the root and shoot fresh and dry weight significantly. The shoot concentration of La decline significantly by 51.53% and increased root concentration by 30.45%.	Hao et al., 2021
Cd, As, and Pb	*Glomus mosseae*	*Pisum sativum*	Inoculation with *G. mosseae* enhanced plant growth, the concentration of carbohydrates, photosynthetic pigments, nitrogen and defense antioxidants. This symbiosis can be employing for onsite remedy of Cd- and Pb-polluted soil.	Chaturvedi et al., 2021
Cr	*Rhizophagus irregularis*	*Brachiaria mutica*	AMF enhanced the photosynthetic performance by increasing the chlorophyll, carotenoid, proline, protein content and activities of antioxidant enzymes. It also improves the tolerance index, transportation index and bioconcentration factor of *B. mutica*.	Kullu et al., 2020
Hg	*Aspergillus* sp. A31, *Lindgomycetaceae* P87*, Curvularia geniculata* P1 *and Westerdykella sp.* P71	*Aeschynomene fluminensis, Zea mays*	The tolerance capacity of plants for the Hg^2+^ was improved after the inoculation of fungi. The biomass of the plants increased along with the reduction in soil Hg concentration. Further, the soil Hg level reduced in *A. fluminensis* by 57.14% inoculated with P87.	Pietro-Souza et al., 2020
As	21 fungal strains including *Humicola* sp.	*Bacopa monnieri*	*Humicola* sp. enhanced the plant growth and bacoside content and can use as a realistic and potential mitigation strategy for reducing the As level in the cropping system.	Tripathi et al., 2020
As	*Piriformospora indica*	*Artemisia annua*	The inoculation of fungi helped the plant to accumulate significantly high concentration of As in roots than shoots. In addition, overall biomass, artemisinin, flavonoids, peroxidase and SOD were increased significantly.	Saeed-ur-Rahman et al., 2020
Cd, Pb, and Zn	*Cenococcum geophilum* (Cg, KY075873.1), *Laccaria* sp. (L1, KY075876.1,), *Pisolithus* sp.1 (P1, KY075877.1*), Pisolithus* sp. 2 (P2, MN422052)	*Pinus sylvestris*	Inoculation of fungi increased the survival rates of plants by enhancing the biomass, photosynthetic rate, transpiration rate, stomatal conductance, mineral nutrients and intercellular CO_2_ concentration. Further, *P. sylvestris* accumulated a higher concentration of Cd, Pb and Zn than non-ectomycorrhizal seedlings.	Liu et al., 2020
As	*Rhizophagus, Funelliformis*	*Pteris vittata*	*Rhizophagus and Funelliformis* inoculation improved the plant growth and increased the fresh and dry weight of aerial parts by 44 and 37%, respectively. The BAF for inoculated plants was 7.6 while for uninoculated it was recorded 6.0.	Cantamessa et al., 2020
Cd and Pb	*Simplicillium chinense* QD10	*Phragmites communis*	The amendments of *S. chinense* QD10 significantly increased the phytoextraction of metal by 28.6–48.0% of *P. communis*.	Jin et al., 2019
Cd	*Acaulospora* *Laevis, Glomus* *monosporum, G. clarum, Gigaspora nigra*	*Trigonella foenumgraecum*	Inoculation of AMF enhanced the plant growth parameters, protein and chlorophyll contents. The TF of plants was also reduced significantly.	Abdelhameed and Metwally, 2019

## Factors Affecting Bioremediation Efficiency

The most important factor affecting bioremediation efficiency is site characteristics. Secondly, environmental factors such as water content, temperature, pH, nutrient availability, moisture content, and pollutant bioavailability can also hinder the efficiency of bioremediation (Freitas et al., 2013; Azubuike et al., 2016; Khodaverdiloo et al., 2020; Leong and Chang, 2020). Apart from this, the bioremediation process is a complex system that is optimized and controlled by many factors. The interactions among the contaminants, microbes, nutrient availability and environmental factors affect the bioavailability and biodegradation of the contaminants.

### Site Characteristics

The first and most important factors which affect the bioremediation process are the site location and its characteristics. The extent and type of contaminants present in the location determine the remediation efficiency (Abatenh et al., 2017). These factors can be overcome and managed by sufficient prior investigation and characterization of sites before implementing the remediation process.

### Temperature

Temperature is an important factor that determines the survival and growth of the microorganism and the composition of hydrocarbon (Yang et al., 2009). It plays a critical role in the microbe-assisted remediation process by affecting both the physical and chemical states of contaminants present in the polluted sites and interrupting the microbial metabolisms, growth rate, soil matrix, and gas solubilities (Megharaj et al., 2011). It is recorded that high temperature destroys the cell metabolic activity of bacteria and affects the process of bioaccumulation (Javanbakht et al., 2014). Furthermore, the temperature can speed up or slow down the remediation process as microbial physiological properties are highly influenced by temperature. The interaction between fungal membrane binding sites and heavy metal ions depends on the temperature. Temperature also affects the configuration and stability of fungal membrane by chemical moieties ionization (Oka et al., 2005). Jin et al. (2019) showed that the biosorption efficiency of *S. chinense* QD10 for Cd and Pb was highest at 30°C by 60.4 and 38.3%, respectively. But it significantly declined when the temperature increased to 45°C. The microbial adsorption is also affected by temperature (Timková et al., 2018).

### pH

pH has its own impacts on the metabolic activity of microorganisms which can increase or decrease the removal process. Bioremediation can be applied in a wide range of pH. However, a pH of 6.5 to 8.5 is considered the maximum potential for remediating the most terrestrial and aquatic systems (Abatenh et al., 2017). The pH value influences the biosorption process by dissociation of functional groups on the fungal membrane and affects heavy metal mobility and solubility (Wang et al., 2014). It was observed that the Cd biosorption capacity of *Exiguo bacterium* sp. enhanced with increased pH up to 7.0 and remained neutral when the pH was higher than 7.0 (Park and Chon, 2016). The microbial adsorption is also affected pH and ionic strength (Timková et al., 2018).

### Nutrient Availability

Likewise, nutrient concentration, availability, and type are also important for microbial growth and activity in the bioremediation process. The fundamental elements (such as carbon, nitrogen, and phosphorous) help the microbes produce the necessary enzymes to break down the pollutants. The lower level of nutrient availability affects the plant and microorganisms, which ultimately affects the bioremediation rate and effectiveness. In this condition balancing the essential nutrient such as nitrogen (N) and phosphorus (P) can enhance the bioremediation efficacy through optimizing the bacterial C:N:P ratio (Abatenh et al., 2017). In the colder environment, the supply of an appropriate quantity of nutrients enhances the metabolic activity of microorganisms, which leads to an increase in the remediation rate (Phulia et al., 2013; Couto et al., 2014). It has been reported that an excessive amount of nitrogen in the contaminated medium resulted in microbial inhabitation (Varjani and Upasani, 2017). Further, the higher concentration of nitrogen, phosphorus, and potassium hinders the biodegradation efficiency of hydrocarbon contaminants.

### Moisture Content

The microorganisms can be adversely affected by the soil moisture content. Moisture affects the rate of pollutant metabolism *via* influencing the amount and type of soluble materials as well as the pH and osmotic pressure of the terrestrial and aquatic sites (Abatenh et al., 2017).

### Type/Nature of Microorganism and Plant

The existence of unsuitable microorganisms or the inadequate presence of suitable microorganisms in the contaminated sites affects the bioremediation efficiency. Apart from this, the microbial biophysical process also influences bioaccumulation as the process is metabolically dependent and uses cellular energy for metal uptake. It depends on the microbial biochemical features, genetic and physiological ability, internal structure, cell surface properties such as charge changes, and surrounding environmental conditions (Srinath et al., 2002; Vijayaraghavan and Yun, 2008; Issazadeh et al., 2013). Razmi et al. (2021) found that phytoremediation efficiency was influenced by various biological and chemical factors. For the plant-based remediation, the important factors consider for selecting the suitable plants includes the root system, it may be tap or fibrous roots depending on the depth of the contaminants, above-ground biomass, which should not preferable for livestock consumption, survival, and adaptation of plants and the plant growth (Azubuike et al., 2016). However, the role of plant type in the phytoremediation of Cd, Pb, Ni, and Zn has been considered as the prime factor. Similarly, the maximum biosorption efficiency for most of the fungal strains was found under their optimal growth conditions (Iram et al., 2015).

### Water Content

In general, microorganisms require water activity values between 0.9–1.0 for metabolism and growth. Most of the bacteria grow optimally at the upper limits of water activity values (Sharma, 2019). Therefore, the water content in contaminated land is an essential factor that may affect the bioremediation rate. Recently, Khodaverdiloo et al. (2020) highlighted that water deficiency, sodicity, and salinity are also important factors that affect bioremediation efficiency.

### Pollutant Bioavailability

The low bioavailability of HMs in the contaminated soil greatly affected the bioremediation efficiency. The bioavailability of contaminants is controlled by various physicochemical processes such as sorption, diffusion, desorption, and dissolution. This problem can be managed using various surfactants and chelating agents, which enhance the bioavailability of HMs for microbial degradation and plant uptake. Various types of organic and inorganic chelating agents are applied recently such as ethylenediamine tetraacetic acid (EDTA), [S,S]-ethylenediaminedisuccinic acid (EDDS), ethylenediamine-di-ohydroxyphenylacetic acid (EDDHA), diethylenetriaminepentaacetic acid (DTPA), nhydroxyethylenediaminetriacetic acid (HEDTA) citric acid, acetic acid, and malic acid. Application of these chelating agents has successfully proven that it effectively forms a complex with HMs and increases the bioavailability (Sarwar et al., 2017).

## Challenges and Future Prospects

The bioremediation methods are diverse and show effectiveness in restoring the polluted sites contaminated with multiple HMs. However, there are some important factors to be considered before implementing bioremediation practices. There is a need for regular investigation and assessment of the level of HMs and other pollutant concentrations in the contaminated sites before proposing bioremediation. The selection of an appropriate type of microbes and plant species is a very hefty task for the sites where the presence of multi-metals and other organic pollutants at the same site. Secondly for the plant-based bioremediation, the presence of volatile metals and metalloids such as Si, Hg, and As in the site may get volatilized into the atmosphere in their toxic form which may affect the living organisms. Third, if edible plants are used for bioremediation purposes, there is a risk that they can be consumed by animals, insects and which may further contaminate the food chain and ultimately reach humans and cause serious health complications. For this, nonedible and nonpalatable phytoremediator plant species can be preferred or in the case of the edible plants, proper protection during cultivation, and harvesting must be taken to avoid future complications. With the presence HMs deeper into the ground where plant roots cannot reach, *in situ* phytoremediation becomes difficult.

Further research, assessment, and investigation are required to enhance our knowledge and understanding of best management practices for efficient bioremediation of HMs. There is a need for futuristic clarification of mechanisms, metabolites, and novel approaches/methods are required. For simple and efficient plant-based bioremediation, utilization of hyperaccumulator plants to efficiently remove of HMs from the contaminated soil need novel strategies for its further progress. This can be achieved in two ways, first by finding and validating the various diversity of new hyperaccumulator plant species, and second by developing the hyperaccumulator plant using genetic engineering. In addition, we can consider the hyperaccumulator plants with deep root plants for, e.g., woody plants or tree such as *Populus × canescens, Rinorea bengalensis, Schima superba* and *Pycnandra acuminata* with high translocation rate, high biomass and growth rates and more tolerant plant species.

Biotechnological intervention including genetic engineering, for example, the rate-limiting step in a known metabolic pathway can be manipulated genetically to enhance the transfer and biodegradation rates, or by introducing a completely new metabolic pathway into the microbe for higher accumulation of HMs or degradation of recalcitrant compounds. In addition, overexpression of foreign genes into a non-tolerant plant with having higher biomass for HM remediation from the soil may be a feasible strategy. The advanced way to study hologenomics of plants microorganism will be helpful for the manipulation of microbial niches which help to enhance the resistance against toxic metal contamination. For multi-metal contaminated and multi-stress environmental conditions, there is a need to development of suitable amendments to enhance the survival of the suitable plant species. Although there are several organic and inorganic amendments and metal chelators are available there is a need for further investigation to find out more suitable and eco-friendly amendments which can be applied for the treatment of multi-metal contaminated and multi-stressed soil. There is a necessity for coordination and contribution of researchers, scientists, policymakers, government, industrial sectors, and individuals that can help to success and reliability of bioremediation.

## Conclusion

Man-made activities have been introducing a high amount of toxic metals into the environment, affecting the life processes of all living organisms in direct and indirect ways. It has been reported that more than one type of heavy metal is simultaneously present in the contaminated land and the available conventional methods are not significantly efficient to detoxify the pollutants compared to the bioremediation process. It has been proved that bioremediation methods are easily affordable compared to other physicochemical remediation techniques. A number of bacterial and fungal strains have been isolated and identified from different metal-contaminated and mining abandoned soils in recent years. *Pseudomona*s spp., *Bacillus* spp., *Aspergillus* spp., and *Penicillium* spp. are found frequently and show high metal tolerance and bioremediation potential. Currently, bioremediation has been practiced in various contaminated sites globally with varying degrees of success. Recently by applying the various plants and microorganisms to remediate the contaminants from the environment has been noted like Alaska oil spill remediation, China’s Aleutian island bioremediation operation and other decontamination cases of HMs from the industrial and agricultural fields. The addition of proper supplements and enhancing environmental conditions are the prime concern for the significant yield of bioremediation. To overcome the above problem, the addition of organic matter and a consortium of microorganisms can enhance microbial metabolic activity and may improve bioremediation potential. In addition, more investigations are still required to screen the more suitable microorganisms, hyperaccumulator plants that will have a high capacity to tolerate multi-metal contaminated and multi-stress environmental conditions sites and accumulate multi-metals at once. Further attention will be required to plant–microbe-based bioremediation strategies to identify the novel plant–microbe pairs that will have high metal removal efficiency along with creating a favorable environment to accommodate other microbial diversity for indirectly improving the soil health. Additionally, further research on the application of nanomaterials and biochar along with microbes to enhance bioremediation efficiency is needed.

## Author Contributions

All authors listed have made a substantial, direct and intellectual contribution to the work, and approved it for publication.

## Funding

This work is carried out at the College of Resources and Environment, Southwest University, supported by the Fundamental Research Funds for the Central Universities (No. SWU 020010), the Natural Science Foundation of Chongqing (No. cstc2021jcyj-msxmX0827) and Chongqing Returned Overseas Students’ Entrepreneurship and Innovation Support Program (No. cx2021001).

## Conflict of Interest

The authors declare that the research was conducted in the absence of any commercial or financial relationships that could be construed as a potential conflict of interest.

## Publisher’s Note

All claims expressed in this article are solely those of the authors and do not necessarily represent those of their affiliated organizations, or those of the publisher, the editors and the reviewers. Any product that may be evaluated in this article, or claim that may be made by its manufacturer, is not guaranteed or endorsed by the publisher.
